# Identification of Legal Barriers to the Rearing and Processing of Insects in the EU—Implications Based on a Case Study

**DOI:** 10.3390/insects17030319

**Published:** 2026-03-16

**Authors:** Jakub Jan Zięty, Elżbieta Małgorzata Zębek, Ewelina Olba-Zięty, Michał Krzyżaniak, Mariusz Jerzy Stolarski

**Affiliations:** 1 Department of Economic Law and Commercial Law, Faculty of Law and Administration, Centre for Bioeconomy and Renewable Energies, University of Warmia and Mazury in Olsztyn, 10-724 Olsztyn, Poland; jakub.ziety@uwm.edu.pl (J.J.Z.); elzbieta.zebek@uwm.edu.pl (E.M.Z.); 2 Department of Genetics, Plant Breeding and Bioresource Engineering, Faculty of Agriculture and Forestry, Centre for Bioeconomy and Renewable Energies, University of Warmia and Mazury in Olsztyn, 10-724 Olsztyn, Poland; michal.krzyzaniak@uwm.edu.pl (M.K.); mariusz.stolarski@uwm.edu.pl (M.J.S.)

**Keywords:** insect rearing, agro-food waste, frass, legal barriers

## Abstract

Insect farming for several purposes, which inscribes itself into circular economy, could become an alternative to traditional agriculture in Europe. Insects are a more sustainable and circular alternative source of protein and fat in food and feeds. The aim of this study is to identify legal barriers to the rearing of insects and marketing of insect-based products. The study focuses on the identification of such barriers to insect rearing and to the production of fertilizers from insect frass. The dogmatic legal method, as well as SWOT and PESTEL analyses, are employed in this research. The two latter methods are used to gain insight into the views held by the industry’s stakeholders. Subsequently, issues within the research field, such as the rearing of insects, their welfare, and the requirements imposed on the feeding of farmed insects, are discussed. Finally, solutions to the identified problems are suggested. The most important strengths of insect farming are its innovative edge and the creation of new products at the EU level. Weaknesses include technological and organizational challenges. Stakeholders attribute high importance to external circumstances, especially economic and social ones. As concluded from this study, the current laws are not optimal for insect farming; however, despite this situation, some changes to the law could facilitate the acquisition of feed for insects or the marketing of some insect-based products. The proposed legal changes aim at lifting the identified barriers to insect farming while still meeting safety requirements and supporting circular economy principles.

## Simple Summary

Insect farming in Europe is evolving into a new branch of agriculture. Interest in the production of insects arises from the search for new, inexpensive and environmentally friendly sources of protein, mostly for animal feeds. Insects have been classified as farm animals; therefore, the legal regulations concerning insect breeding and rearing are based on the laws governing farm animals, which gives rise to problems in some areas. The purpose of this paper is to identify the legal regulations that hinder the development of insect farming and manufacture of insect-based products, and to suggest changes to existing laws. The identification of such barriers was achieved against the backdrop of external and internal factors influencing the growth of this sector. The study allowed us to identify two legal problems related to these areas: (1) the approval of feeding insects with residues from agriculture, the agricultural and food processing industry, as well as delicatessen and catering by-products and waste; and (2) the conditions for processing insect frass for the fertilization of agricultural and horticultural crops, and the approval of the sale of frass. It was concluded that by expanding the inventory of acceptable products from agricultural or food waste (catering waste) for feeding insects, it would be possible to reduce costs of waste management in agriculture and in the catering industry. Regarding the second problem mentioned, it was found that expanding the catalogue of methods for the preparation of frass and allowing frass to be handled in the same way as animal production residues are managed could significantly reduce costs and enable entrepreneurs to optimize technologies for the production of fertilizers from insect excreta.

## 1. Introduction

Insects have long been used for the production of food and feed in Asia, Africa and South and Central America. The consumption of insects (or entomophagy) is part of the regular diet of many Asian, African, and Latin American cultures. Insects are recognized as a valuable source of bioactive compounds (bioactive peptides, chitin and chitosan, phenolic compounds, and fatty acids) and provide health benefits (antioxidant, antihypertensive, anti-inflammatory, antibacterial, and immunomodulatory benefits). Therefore, edible insects are considered a good source of nutrients when consumed as human food, but they can also be used as ingredients in nutraceuticals and functional foods [1,2]. Insect farming is considered a potential resource to help address food security challenges by 2025. In addition to the dietary benefits mentioned above, it also significantly improves production sustainability through reduced environmental impact. This has led to greater interest in insects as an alternative protein source not only for food production but also for animal feed [3]. For example, in farm animals (poultry and pigs), insect feed can replace soybean meal [4]. Previous research has indicated that a sustainable agri-food system should be economically viable and efficient, but also ecologically sustainable and socially responsible towards farmers, workers, communities, consumers and society [5,6]. Heavy metals are a potential cause of food contamination, which can threaten food safety [7]. Therefore, insects require commercial processing methods that make the protein suitable for food and feed formulations while maintaining the safety, nutritional, and sensory value of the final product. Commonly used methods include lipid extraction, enzymatic proteolysis (enzymatic proteolysis), commercial thermal processing (e.g., blanching, pasteurization, and commercial sterilization), low-temperature processing (chilling and freezing), dehydration, and fermentation technologies. Each of these methods has its advantages and disadvantages, which must be carefully considered, as not all processing methods and/or conditions apply to all edible insects or insect flours [8]. The global food system is currently undergoing transformation. New protein sources are gaining importance in response to growing pressures on agricultural land, climate stability, and food security. Among these alternatives, edible insects have become a strategic resource owing to their high feed conversion efficiency, smaller environmental footprint, and rich cultural heritage in various regions of the world [3,9]. While there are technologies currently available for breeding, producing and manufacturing protein and other products from insects, their development is dependent on the existence of a regulatory framework capable of ensuring safety, standardization and consumer confidence [9]. Many countries across the world, through their administrative bodies for food matters, regulate the range of insects approved for the purposes of nourishment [10,11,12]. China has national food diversification strategies in place to address the growing interest in functional foods derived from insects. Research is underway on new insect species for future production [12]. In Latin America, regulations regarding edible insects remain fragmented and usually lack a specific regulatory framework [9]. Thailand, an example of a country with a growing insect sector, supports the development of this branch by the Bio-Circular Green (BCG) strategy, designed and implemented for this purpose. This will enable Thailand to transition from traditional harvesting to an export-oriented, innovation-driven insect bioeconomy, positioning the country as a benchmark in the Asia–Pacific region for insect management and supply chain sustainability [9,11].

Meanwhile, the European Union member states consider insect farming as a new element of agricultural production and a novel source of food or feed, which constrains the use of insects [13,14]. In consequence, such matters as the rearing and slaughter of insects or production of protein and other insect-based products are regulated by general laws, which apply to all farm animals and only slightly address the specificity of insects by passing targeted solutions. The lack of precise legal regulations means that insect use and rearing develop differently in individual EU member states. Bearing in mind the specific nature of insect farming, both producers and authorities must apply good practice guidelines for insect farming, which are regularly updated. The application of regulations that concern conventional livestock farming practically hinders the growth of this new branch of agriculture [15]. An area where existing legal solutions might be amended for the sake of insect rearing is the determination of maximum permissible thresholds of various chemical and microbiological hazards to these invertebrates [15]. Currently, regulations have become a decisive factor shaping the future of the insect-based food sector. In regions such as the European Union, the establishment of legal definitions, authorization pathways, and hygiene standards has enabled the emergence of structured value chains, export-oriented businesses, and innovation ecosystems supported by investor confidence. In comparison, some regions with long-standing traditions of entomophagy (Latin America) are only just moving toward harmonized policies, recognizing edible insects as legal components of the formal food system [2,3].

There are research papers dealing with the question of insects as a source of protein for human food and animal feeds [14,16,17,18]. Some researchers have also observed the issues arising from the EU law in the context of insect farming in the European Union [15,19,20,21]. The cited authors focused on general issues and did not undertake to identify in detail such specific barriers as those preventing the use of catering waste for insect nourishment or the processing of frass for fertilizer production. These two research problems represent the novelty aspect of our study and, more importantly, the results achieved fill in the above gap in knowledge as well as add new information to some review papers published previously.

The aim of this study is to identify and implicate the legal restrictions concerning the use of substrates originating from insect farming. The authors consider the rearing of insects to be an opportunity to close the supply chain by feeding insects with catering waste, which would otherwise have to be deposited on landfills. Furthermore, insect frass could be a source of additional income if used to make fertilizers or soil amendments. To this aim, the applicable legal regulations governing insect farming, and the conditions allowing for the use of frass for fertilization are reviewed. In addition, the authors suggest possible changes to law that could make insect farming easier.

## 2. Materials and Methods

This study was divided into several parts, each devoted to a specific scope of problems related to insect rearing. The first part presents the results of an SWOT analysis of the growing insect industry in Europe [22]. The SWOT analysis was applied to evaluate both internal and external factors. Four groups of factors, i.e., strengths, weaknesses, opportunities and threats, were analyzed [23]. The strengths and weaknesses comprised internal factors, while the opportunities and threats referred to the factors external to insect farmers and insect-based product manufacturers [24]. The analysis was carried out on the data obtained during a workshop discussion with a group of 55 workshop participants from Poland, who represented insect breeders (14), insect breeding advisory and supervision bodies (9), insect-based product manufacturers and sellers (5), and scientists working on insect rearing and insect-based product manufacturing technologies (20). An important group of respondents consisted of lawyers specializing in economic and commercial law (7).

The next step of the analysis consisted of the assignment of the identified factors according to the methodology of strategic analysis assessing the macroeconomic environment. The method itself is known as the PESTEL approach, an acronym of the six groups of factors mentioned [24], and in accordance with its assumptions, the political, economic, social, technological, environmental and legal factors were analyzed. The evaluation of the factors was based on the following description of each group [25]. Political factors, denoted with the letter P, were the ones that affected the organization through international, national, regional and local policies, either existing or being developed. Consequences of political factors could be seen in business plans, instability of policies, competition policy, policy of incentives and subsidies and the approach to commerce and customs. Economic factors (E) concerned the overall condition of economy, including costs, inflation, interest rates, economic growth, and currency exchange rates, as well as geographical factors influencing the economic environment. Social factors (S) included such long-term factors as a population growth and age demographics, and short-term ones, i.e., social trends and shopping pattern changes. Technological factors (T) concerned the range of technological change, R&D and automation in the industry’s direct operation and in the chain of supply it uses. Technological development also referred to the production and operational practice and to the product or service itself. The assessment of technological development also included factors interfering with its traditional course. Environmental factors (En) were included in the evaluation of the impact of the industrial branch on the environment. Both positive and negative effects were considered. Legal factors (L) focused on the legal regulations directly governing the company and its area of activity, both on the domestic and on international markets.

The subsequent part of this article presents the legal status of insect processing (Section 3.2), and then the rearing of insects for animal feed (Section 3.5) and insect welfare (Section 3.6), as well as the use of frass for fertilization purposes (Section 3.7). The following sections contain a report on the problems most often pointed to by stakeholders (Section 3.7) and the identified legal problems affecting insect farming and the marketing of frass-based fertilizers (Section 3.8). The last section presents some suggestions of necessary amendments to legal solutions that could facilitate insect farming and production of fertilizers from insect frass (Section 3.10).

This stage of the study that intended to identify legal obstacles to insect farming was also based on a workshop discussion with stakeholders, as described earlier. The outcome was the identification of legal problems and a proposal of legal solutions.

The basic methods used for legal analyses included the dogmatic legal method and the comparative method. The former takes the results of the linguistic (grammatical), systemic, and teleological interpretation into consideration. It was applied to analyze the legal acts relevant to the research problem. The linguistic interpretation was carried out mainly in relation to the provisions introduced in the Directive and Regulation. In addition, it was necessary to refer to the regulations concerning the manufacturer’s responsibility. The outcome of the analysis supported by the dogmatic method served as a starting point for the next stage of the research, where the legal comparative method was employed.

## 3. Results and Discussion

### 3.1. An SWOT Analysis of the Insect Farming Sector

**Figure 1:**
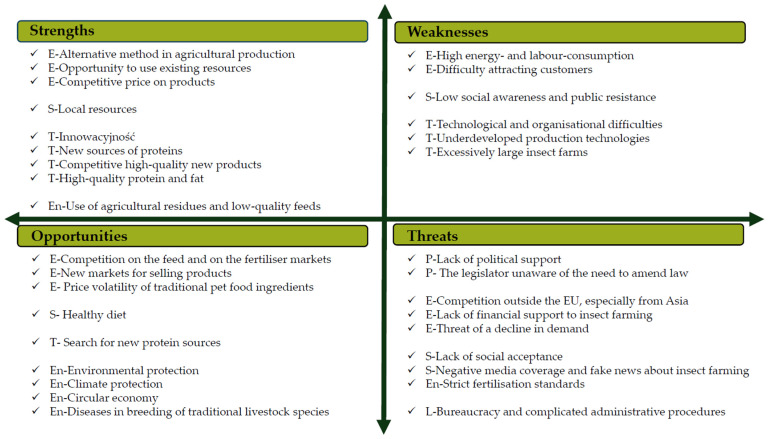
An SWOT-PESTEL analysis for the insect production branch in Poland.

An SWOT analysis was carried out with the participation of people involved in insect farming and in the marketing of insect-based products. It revealed both strengths and weaknesses of insect production. Most strengths were related to technology, where the innovative edge of insect production was most often mentioned (Figure 1). It was also underlined that insect-based products are a new source of high-quality protein and fats. Strengths were also identified in connection to economic factors. Insect production was mentioned as an additional source of income from an alternative agricultural activity; furthermore, existing production resources can be employed, and there is a chance to price insect-based products competitively. Weaknesses were also observed, and, again, technological factors comprised the largest group. Mostly, the respondents pointed to technological and organizational difficulties, and production technologies being still imperfect. Among the economic factors, which created the second largest group of factors identified as weaknesses, high energy and labor consumption of production, which corresponded to cost-intensive production, were most often implicated. Also, difficulty attracting customers was identified as a weakness.

Economic factors, seen as either opportunities or threats, were most numerously mentioned among the external factors that affected insect production. Opportunities mainly concerned offering new products on the animal feed and pet food markets, whereas limitations (threats) were related to competitive products from outside the EU, especially from Asia, a decline in demand, and a lack of support for this new, developing branch of economy. Among the opportunities, the ones most often mentioned were the positive impact of the branch on the environment, especially its protection, and a contribution to the growth of circular economy. Social factors were most often identified as threats and included the lack of social acceptance for insect farming and insect-based products, as well as negative and fake information about insect farming disseminated in the media among the public. Other limitations were identified in the areas not mentioned previously, i.e., the legal conditions. Bureaucracy and complicated legal regulations, often unadjusted to specific characteristics of insect production, were seen as threats. An SWOT analysis, especially when combined with a PESTEL analysis, establishes a solid foundation for the identification of needs and fortification of the insect production sector, as demonstrated by similar analyses made earlier for other industries [11,24,26,27,28,29].

### 3.2. The Legal Status of Insect Processing

The study analyzed the following areas of legal regulation: (1) insect rearing, (2) feeds for feeding farmed insects, (3) insect production for feed, (4) welfare of insects, and (5) frass as fertilizer (Table 1). Issues related to the processing of insects regulated by Polish law are presented in Table 2.

### 3.3. Legal Conditions Concerning Insect Rearing

In practice, until 2018, the concept of edible insects as a food product had not been specifically regulated in the European legal system. This issue was left to national legal systems. The EU member States independently decided how to regulate the breeding, processing, and manufacturing of these products. The production of insects for consumption was possible in accordance with the general principles of food law. Consistent with the precautionary principle, it was necessary to identify potential hazards associated with novel food products, conduct a risk assessment, and develop interim risk management measures. The introduction of insects as novel foods was permitted under general principles [53].

The overall legal regulations concerning food and feed safety, which also apply to insect rearing, include Regulation (EC) No 178/2002 [30], and Regulations (EC) No 852/2004 [31], 853/2004 [32] and 2017/625 [33] (Table 1). One of the specific requirements laid down in Regulation (EC) No 852/2004 [20] is the provision that any enterprise in the food industry must implement and maintain a procedure or procedures based on the Hazard Analysis of Critical Control Points (HACCP). In principle, the use of an HAACP system is not mandatory in primary production of animal origin food (agriculture), which is governed by more detailed requirements laid down in Regulation (EC) No 853/2004 [32] (Table 1). However, this document does not contain any clauses regarding insect farming; an amendment aiming to address this branch of farming was proposed in 2019, but it was annulled in 2022 (Document Ares (2019) 382900) [54]. Most activities related to insect farming comprise both the rearing of insects and their processing [“slaughter”], whereas the HACCP provisions apply to the processing of raw materials (secondary production). The application of the HACCP principles to insect rearing and processing should, therefore, be considered a prerequisite in practice. This approach involves the identification of any possible hazards that must be prevented, eliminated or reduced to acceptable levels, and the establishment of critical control points, which are needed to prevent, eliminate or reduce such hazards to acceptable levels. An alternative way to comply with the above requirement is to adhere to a national and an EU good practice guide. Such a guide was developed in 2022 by the International Platform of Insects as Food and Feed (IPIFF) [55], and approved of by the Standing Committee on Plants, Animals, Food and Feed of the European Commission on Plants, Animals, Food and Feed (SCoPAFF) [56] concerning “the biological safety of a food chain”, and the SCoPAFF section responsible for “animal feeding”. The guide is publicly available; however, full compliance with all applicable regulations may call for more specific implementation and/or further steps, depending on particular operations and deviations from the processes described in the guide. The general regulations on food labeling were specified in Regulation (EC) No 1169/2011 [34] (Table 1). Subsequently, more general requirements were laid down in Regulation (EC) No 767/2009 [35] (Table 1), which prohibits the use of some materials, for example, household waste or manure, for animal nutrition (Annex III). It is worth noting that these general requirements do not exhaust the question of food and feed safety, but a thorough review of laws regulating these issues and applicable to all food and feed production establishments is beyond the scope of this study.

### 3.4. Products (Feeds) Allowed for Feeding Farmed Insects

At present, not all products are approved for insect feeding. In the EU, insects intentionally reared by people are considered “farmed animals”, in line with the definition contained in Article 3, point (6) of the Regulation of the European Parliament and of the Council (EC) No 1069/2009 [36]. The definition of farmed animals includes (a) “any animal that is kept, fattened or bred by humans and used for the production of food, wool, fur, feathers, hides and skins or any other product obtained from animals or for other farming purposes; (b) equida.” This legal classification means that insects are submitted to the feed ban regulations like any other farmed animals. Insects can only be fed materials approved for animal nutritional purposes, that is, plant or animal materials which are mentioned in Annex IV to Regulation (EC) No 142/2011 [37] and Annex XIV Chapter 1 Section 2, 5b of Regulation (EC) No 999/2002 (Annex IV) [38]; Regulation (EC) No 1069/2009 of the European Parliament and of the Council [36] (a ban on the use of manure, catering waste and untreated animal by-products as substrate for animal rearing); Regulation (EC) of the European Commission No 142/2011 [37]; Regulation (EC) of the European Parliament of the Council No 999/2001 [38] (introduces a feed ban—Article 7 and Annex IV); Regulation (EC) of the European Commission No 68/2013 [39] (a catalogue of feed materials; not all materials are permitted for insect farming); Annex III of Regulation (EC) of the European Parliament and of the Council No 767/2009 [35] (materials prohibited in animal nutrition, including urine and feces, hides, packaging materials, wood), and Regulation (EC) of the European Parliament and of the Council No 1831/2003 [40] (authorized feed additives) (Table 1).

Currently, food producers, wholesalers and retailers supplying insect farmers with plant-based substrates (i.e., food no longer intended for human consumption, but for the use as animal feed or obtained in the process of food production, or an end food product) are legally obligated to adhere to the EU law governing feed hygiene (cf. The European Commission Guidelines concerning the feed use of food no longer intended for human consumption) unless insect producers process the product further in compliance with the EU rules on feed hygiene (e.g., an implemented HACCP system). According to Section 10 of Annex X to Regulation (EC) no 142/2011 [37] (Table 1), food containing milk, milk-derived ingredients, eggs, egg products, honey, rendered fats, collagen and/or gelatine and removed from human consumption can be used directly (that is with no further processing) by insect farmers on condition that these products have been processed in compliance with the EU regulations concerning food hygiene and that they originate from the EU. Impurities or harmful substances cannot exceed the threshold amounts envisaged in Directive 2002/32/EC [41] on undesirable substances in animal feed (Table 1). The regulations permit the following animal-based products to be used as substrate in insect farming: fish meal, fish oil, products from the blood of animals other than ruminants, hydrolyzed proteins from ruminant hides, gelatine and collagen, eggs and egg products, milk and milk-based products, colostrum, honey, and rendered animal fats. With regard to insect feeding, the regulations envisage one exception. Insects intended as fishing bait can be fed catering waste or animal manure (Category 2 material) provided a competent authority authorizes it in accordance with Article 18 of Regulation (EC) no 1069/2009 [36] (Table 1). The regulations do not permit the use of waste for feeding purposes. However, should any waste be classified as a by-product, then its use for animal nourishment would be allowed. In the EU law, in accordance with Article 5 of Directive No 2008/98/EC [42] on waste (Table 1), for a substance or object resulting from a production process not to be considered a waste but a by-product, all the following four conditions must be met: (1) further use of the substance or object is certain; (2) the substance or object can be used directly, without any processing other than regular industrial practice; (3) the substance or object is obtained as an integral part of a production process; and (4) further use of the substance or object is lawful, i.e., the substance or object meets all applicable product, environmental or health requirements, and will not cause overall adverse environmental and human health impacts. These regulations were implemented into Polish law in Article 10 of the Act of 14 December 2012, on waste [48] (Table 2). Thus, unlike waste, by-products must meet the above requirements, which means that many substrates of plant and other origin that might be used for insect feeding cannot satisfy these requirements. Only animal-origin by-products classified as Category 3 materials or products originating from such animal-origin by-products other than Category 3 materials, mentioned in Article 10 n), o) and p) of Regulation (EC) No 1069/2009 [36] (Table 1), can be used for production of processed animal protein. Therefore, allowing catering residues or catering waste for insect feeding could help expand the supply chain. In addition, permitting the use of agricultural production residues could complement the supply chain available to insect-rearing enterprises. In the literature, restrictions on the use of some substances for rearing insects (insects can only be grown on plant-derived substrates or by-products from animals mentioned in Annex X of Regulation No 142/2011 (EC) [37] (Table 1), which include milk and milk-based products, eggs, etc.) are considered a barrier to the growth of insect production [57]. It is crucial to expand the range of allowed substances to maximize the potential of insect production and to improve the sustainability of the food/feed chain [15,58,59,60].

### 3.5. Insect Production for Feed

#### 3.5.1. Types of Insect-Based Products for Feeding Other Animals

Insect-based products intended to be fed to other animals can be generally divided into four categories: (1) live insects, (2) processed animal proteins (PAPs), (3) fat, and (4) chitin and other derived products. From the legal perspective, the species of an insect and the genus of an animal that is to be fed insect-based feeds are most important. The legal ramification for insects as feed specifically defines the allowed insect species, while the categorization of farm animals is based on a higher taxonomic level or comprises several genera or families (poultry, ruminants, etc.), without specifying species [15,27].

The production of insects for feed purposes is currently regulated by the EU law, and only to a limited extent by national regulations (in this case, those of Poland). EU legislation consists of general regulations applicable to all types of animal production or the production of feeds. They relate to insect production only to a small degree. General regulations take little account of the specific character of insect rearing or processing, which has an impact on production costs as well as the possible use of resulting products. The lack of specific regulations applicable to insect production is partly addressed through good practice guidelines, monitoring procedures or the implementation of HACCP protocols.

Live insects have long been used in the EU as fish bait or animal feed [61,62,63,64]. In February 2024, the SCoPAFF clarified that “live insects, considered as feed materials, may be legally used as feed in the EU, except for ruminants (restriction due to the feed ban laid out in Article 7(1) of Regulation (EC) No 999/2001 [38] (Table 1), within the general Union legislative framework for feed. This includes, in particular, compliance with the general safety and marketing requirements laid down in Article 4 of Regulation (EC) No 767/2009 [35] (Table 1), to be examined by the competent control authorities on a case-by-case basis” [56]. Thus, other than as animal feed, insects reared to be sold as live insects can also be fed to more conventional livestock raised for food, such as swine, poultry or fish in aquaculture.

Another category of insect-based feed consists of processed animal proteins (PAPs). PAPs are animal proteins derived from Category 3 material, which were treated in a specific way to be suitable for feeding to other animals or as a fertilizer. Fats intended to be fed to other animals can be rendered from Category 3 materials except animal hinds and catering waste (Articles 10 and 31 of Regulation (EC) No 1069/2009 [36], Annex X of Regulation (EC) No 142/2011 [37] (Table 1). There are no restrictions on which animals, including ruminants, can be fed these fats. With regard to rearing insects, the same regulations apply to production of hydrolyzed proteins. Detailed requirements concerning the processing of fats and hydrolyzed proteins are contained in Annex X of Regulation (EC) No 142/2011 [37] (Table 1).

Some animal-derived products like milk and eggs are excluded from the above definition and do not require the same degree of processing as needed for production of PAPs (Regulation (EC) No 142/2011, Annex I) [37] (Table 1). Category 3 in this definition refers to the three categories of animal by-products mentioned in Regulation (EC) No 1069/2009 [36] (Table 1). Category 1 creates the highest risk of bovine spongiform encephalopathy, whereas Category 3 materials can be considered the least risky in this regard. The processing of insect biomass to PAPs is only allowed when producers use one of the five methods listed in Chapter III of Annex IV of Regulation (EC) No 142/2011 [37] (Table 1). Each of these methods entails specific requirements concerning the particle size of animal by-products to be processed, the temperature reached during the processing, and the pressure and duration of the processing treatment. Alternatively, producers may apply for a modified processing method to be authorized by the competent authority (as processing method 7). In this case, the applicant must identify all relevant hazards and demonstrate the method’s capacity to reduce these hazards to a level that does not pose a significant threat to public and animal health. Furthermore, the end-product must be sampled on a daily basis for 30 days to verify its compliance with the microbiological standards set for *Salmonella*, *Enterobacteriaceae* and *Clostridium perfringens*.

At present, eight insect species are allowed to be processed to PAPs: two species of flies (*Diptera*), two species of beetles (*Coleoptera*), three species of grasshoppers and crickets (*Orthopthera*) and one butterfly (*Lepidoptera*). Orthoptera undergo only partial metamorphosis (egg, nymph, adult/imago), while the second order undergoes complete metamorphosis (i.e., pupation); although it can be assumed that holometabolic insects tend to be harvested in the larval stage, the use of adults does not seem to be explicitly prohibited. In 2021, silkworm (*Bombyx mori*) was added to the original seven insect species approved for farming in 2017 following Regulation (EC) No 2017/893 [43] and Regulation (EC) No 2021/1925 [44] (Table 1). This decision was justified by referring to the risk profile developed in 2015 by the European Food Safety Authority (EFSA) and a brief discussion on the nature and safety of this insect if used as feed. Other insect species were also mentioned in the EFSA report as “having the highest potential for food and feed” and at some point possibly permitted. These are *Zophobas atratus* (giant mealworm), *Galleria mellonella* (greater wax moth), *Achroia grisella* (lesser wax moth), *Locusta migratora migratorioides* (African migrating locust) and *Schistocerca Americana* (American grasshopper)—but none of them has been assessed yet; therefore, the inclusion of these (or other) species remains speculative. In contrast to the legal ramification of food production (described in Section 3 below), there is no formal and published procedure of adding new species to the current list of eight.

#### 3.5.2. Regulation of Feed Production

The scope of activities pursued by business entities operating on the feed market is regulated at all stages, from primary production to marketing, by Regulation No 183/2005 [45] (Article 2) (Table 1). The regulation specifies overall conditions and measures enabling the traceability of feed, as well as conditions and procedures for the registration and establishment of feed companies. The establishment and maintenance of insect farming must be preceded by satisfying several requirements concerning the proper preparation of a site for rearing as well as the choice of an insect species to be reared. Formal requirements apply as well, such as registering as an insect breeder or obtaining a decision on entry into the register of animal breeding entities. Insect farming is regularly monitored. The system of registration and approval of all feed companies is a significant element of the monitoring procedure because it is a means of effectively tracing the route of products, from their manufacturer to the end user. It also facilitates effective official controls. The system of issuing approvals for the operation of feed companies in particular should take into consideration the activities that create higher risks in the feed manufacturing process. In order to be approved or registered, feed companies must meet numerous requirements related to their activities, facilities, equipment, personnel, production, quality control, storage, paperwork, etc., so as to ensure both feed safety and traceability of products. It seems that there should be an option of adjusting these requirements to different types of feed companies. The feed companies already operating on the market underline the complexity of the procedure applicable to insect rearing and insect processing to feeds. In Polish law, the provisions of the Act of 6 March 2018, Entrepreneurs’ Law (Article 6 item 1 point 1) [49] (Table 2) do not apply to such types of agricultural activities as crop production, livestock rearing, horticulture, vegetable growing, forestry and inland fishing. In consequence, a business activity consisting of the rearing and production of useable insects on a farm is not subject to entry in the Central Register and Information of Economic Activity (CEiDG). However, before starting insect farming, one needs to contact the district veterinary doctor in order to be issued relevant decisions and required entries in the relevant registers. The procedure for the registration of business entities and the obligation to report to the district veterinarian such information as the scope and size of production, types of products to be produced in a business entity is stipulated in the provisions of the Act of 16 December 2005, on products of animal origin [50] (Table 2). A written application for entry into the register of companies must be submitted to the district veterinary doctor at least 30 days in advance of the onset of the planned production. Such registered business activity can be conducted after obtaining an administrative decision on entry into the register of companies.

#### 3.5.3. Law Regulating Feed Production

Any company operating on the feed market is also obligated to comply with the general rules of food hygiene and to implement an HACCP system, as laid down in Regulation No 183/2005 [45] (Table 1). With respect to producers of insects for feed at “other stages than primary production”, i.e., from the stage of slaughter to subsequent processing stages, they must comply with detailed hygiene requirements set out in Annex II to the mentioned regulation. These requirements detail the conditions of facilities and equipment, personnel, storage and transport, mandatory sampling plans, record-keeping procedures, complaints and product recalls. Insect-based feed manufacturers must also apply the methods specified in Regulation (EC) No 142/2011 [37] and are obligated to choose between processing methods 1 to 5, or method 7, referred to in Annex IV to the mentioned regulation (Table 1). Businesses operating on the feed market must ensure that all the production, processing and distribution stages they carry out and control comply with the EU law, national law compliant with the EU law, and good practice guides. In particular, they must make sure that all relevant hygiene requirements set out in the mentioned EU regulation be met. When feeding animals intended for food production, farmers use means and procedures whose aim is to keep biological, chemical and physical risks of any contamination of feeds, animals and animal-derived products on the lowest possible level. Business entities operating on the feed market are also obliged to introduce, implement and apply on a daily basis a written procedure based on the HACCP principles. It comprises the following stages: (a) identification of all hazards, (b) determination of critical control points, (c) determination of threshold values of the critical control points, (d) development and implementation of effective procedures for the monitoring of critical control points, (e) establishment of corrective measures, (f) establishment of verification procedures, and (g) maintaining records. However, it should be mentioned that it is impossible to identify critical control points in some feed companies, and in some cases good practices may replace the monitoring of critical control points. Feeds sold on the market should meet the requirements set out in Regulation (EC) No 767/2009 [35] and in Article 15 of Regulation (EC) No 178/2002 [19] (Table 1). First and foremost, feeds must be safe, and they cannot have a direct adverse effect on the environment or on animals’ welfare. Moreover, feeds must not be spoilt, altered or adulterated, and they must be suitable for the intended purposes and of fair commercial quality, properly labeled, packaged and presented in accordance with the provisions of Regulation (EC) No 178/2002 [30] (Table 1). Thus, the numerous procedures and requirements that feed producers and traders must comply with (approval, registration, feed hygiene requirements, HACCP and monitoring) makes such business activity significantly more complex and administratively challenging. It is, therefore, worth considering if the complexity of applicable procedures should not be relaxed.

### 3.6. Welfare of Insects

The question of welfare in animal rearing is receiving increasing attention. This aspect was harmonized in Directive 98/58/EC [46] (Table 1). However, invertebrate animals except live cephalopods are excluded from the directive (Article 1). In consequence, according to the regulations, the producer does not need to take care of “insect welfare”. On the other hand, relevant regulations can be entered into force in the legal systems of individual member states. For example, such solutions have been implemented in the Netherlands. General legal regulations applicable to insects mandate that insects should be kept free from hunger and thirst, discomfort, pain, injuries or diseases, fear or stress; instead, they should be able to express their natural behavior [65]. The question of how these five freedoms should be interpreted with respect to insects is a subject of ongoing debate, but some research papers published recently have provided us with an initial framework; it is unclear if farmed insects should be considered aware creatures, but there is a consensus that a precautionary approach to insect welfare should be employed [66,67,68,69]. The rearing conditions that optimize production efficiency in terms of survivability and efficiency for commercial purposes are most probably compliant with welfare requirements, but also specific for a given species. A more general welfare aspect is the method used to sacrifice insects. One of the original barriers to using insects as feed for aquaculture was the fact that animal-derived by-products used for production of feeds for animals (PAPs) had to originate from abattoirs that had detailed protocols on the welfare and slaughter of animals. However, the nature of insect farming made it impossible to adhere to the provisions of Regulation (EC) 2017/893 [43] (Table 1). This mandated “abattoir” condition has been, therefore, abolished in the case of insect farming, but new EU regulations pertaining to the welfare of insects before slaughter have not been developed yet. The IPIFF has published an information sheet dealing with insect welfare and aiming to create more uniform industrial practices, but this document is not legally binding and lacks legal force [55]. Problems of insect welfare have been raised in the literature [15,66].

### 3.7. Frass as Fertilizer

Another element in the new supply chain could consist of using insect feces (frass) for fertilization. The potential application of frass as fertilizer arises from its content of nitrogen, phosphorus and potassium in amounts comparable to those in other natural fertilizers, like FYM [70,71,72,73,74]. Earlier studies proved that frass can also have a comparable content of nutrients to that determined in manure of mineral fertilizers [70,71,72]. The available research results have shown that application of frass (especially from black soldier fly) for fertilization can bring about promising yields, comparable to ones achieved with other fertilizers, in cultivation of chili peppers [73] and maize [74]. Both studies showed that frass contained mainly nitrogen, phosphorus and potassium.

Law allows for the possibility of using frass for fertilization. According to Annex I to Commission Regulation (EU) No 142/2011 [37] (Table 1), insect frass is a mixture of feces from farmed insects, nutrient substrate, parts of farmed insects and dead eggs, with the content of dead farmed insects not exceeding 5% of the volume and 3% of the mass of frass. If the amount of dead insects is higher than 5%, then the material is no longer defined as frass and, in accordance with the European Commission’s opinion, should be processed by pressure sterilization. Every insect producer bears the responsibility for considering the characteristics of the insect species they rear and designing a process of their harvest that would enable successful separation of larvae or adult specimens from their excreta, dead insects and remaining substrate. Most insect producers use larval screens, or “sorters”, or the manual picking-up of insects. This sorting helps to detect and remove foreign bodies (e.g., metals or plastics from the equipment) effectively. Frass must meet the requirements set out in Annex I and Annex XI to the mentioned regulation. Frass must originate from an enterprise making derived products for use outside the feed chain, or from a biogas or composting plant, or from a plant producing organic fertilizers or soil improvers. For frass to meet the safety requirements defined in Regulation (EU) 2021/1925 [44] (Table 1), it must undergo sterilization by thermal treatment at 70 °C, and the minimum treatment duration is 60 min without interruption. The reason is that frass must meet microbiological standards regarding the presence of *Escherichia coli* and enterococci. In compliance with the cited regulations, no alternative processing is allowed. However, the permitted technology is very expensive and labor-consuming, while other sterilization technologies can yield similar outcomes. A question, therefore, arises as to whether the EU legislator might introduce other alternative and economically more feasible frass sterilization methods. For frass to be considered insect-based natural fertilizer, it must also possess certain properties. Research has shown that frass can be a valuable raw material for the production of fertilizers [75], improving the fertility of soil [76]. The legal regulations governing this aspect of insect farming are as follows: (a) Regulation of the European Parliament and of the Council (EU) No 2019/1009 [47] laying down rules on the making available on the market of EU fertilizing products (Table 1); (b) The Act of 10 July 2007 on fertilizers and fertilization [51]; c) Regulation of the Minister of Agriculture and Rural Development of 9 August 2024 on implementation of certain provisions of the Act of Fertilizers and Fertilization [51] (Table 2). These regulations specify, for example, the competent research entities as well as minimum quality standards that fertilizers or crop production amending substances must meet. With respect to fertilizers, it is mandatory to determine their impact on humans, animals and the environment. In Poland, the Rural Medicine Institute is responsible for making assessments of the impact on human health, the State Veterinary Institute issues opinions regarding animal health, and the Institute of Environmental Protection evaluates effects on the natural environment. The most important criteria applied to evaluate organic and organic-mineral fertilizers are the lowest allowable levels of contamination with heavy metals, i.e., chromium, cadmium, nickel, lead and mercury. In addition, frass-based fertilizers must not contain any live eggs of intestinal parasites of *Ascaris* sp., *Trichuris* sp., *Toxocara* sp. or bacterial cells of the genus *Salmonella.* Fertilizers must also satisfy the minimum quality standards with respect to the content of nutrients, i.e., nitrogen, phosphorus and potassium. In Poland, organic and organic-mineral fertilizers are assessed in terms of the minimum quality standards by the Institute of Soil Science and Plant Cultivation - State Research Institute (IUNG - PIB). It is mandatory to obtain a marketing permit for a fertilizer or crop cultivation amending product when introducing new organic, organic-mineral, mineral fertilizers and amending substances. The Minister of Agriculture issues a decision on allowing the marketing of a fertilizer or crop production amending product, having obtained positive opinions of authorized organizational units, supported by laboratory tests confirming that a fertilizer or a product meets quality standards. Not all fertilizers require permits. With regard to frass, research confirms that this material possesses the properties that will enable it to obtain a positive opinion about its fertilizing potential. The registration of a fertilizer produced from frass would create an opportunity for insect producers to earn an additional income.

Problems related to frass production and utilization are not limited to the European Union. The USA and Canada also struggle with such legal issues. An example of a legal problem in Canada is the exclusion of insects from the list of manure-producing animals. In the USA, production of protein from insects or the use of insect farming by-products such as frass as fertilizer or soil amending substance are regulated by federal, state or local regulations [77].

### 3.8. Problems Indicated by Stakeholders Regarding Insect Farming and Frass Production

**Figure 2:**
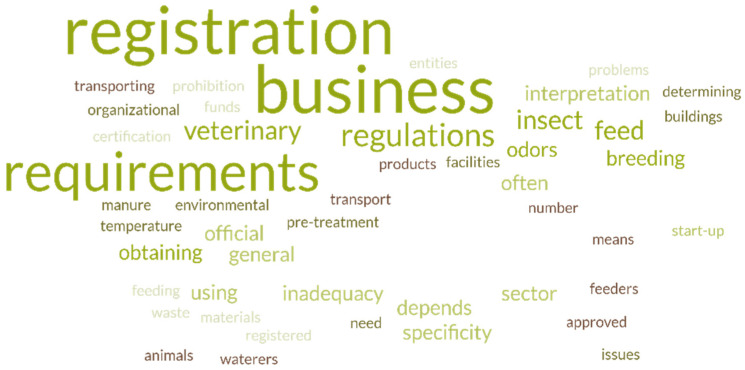
Legal problems identified by a group of stakeholders engaged in insect farming in Poland.

Having analyzed the perception of legal problems by a small group of people involved in insect farming in Poland, several insights were gained regarding the general challenges that appear in such production. As mentioned in Materials and Methods, the problem that the surveyed stakeholders indicated most often was the registration of an insect farming and processing business activity, as this was considered difficult by 10 respondents (Figure 2). Other problems mentioned more than once were veterinary requirements, odors, requirements imposed on insect farming, feed requirements set out for insect nourishment, overall lack of adjustment of law to the specific nature of insect breeding, and the interpretation of regulations oftentimes dependent on an official. Moreover, there were questions like difficulty sourcing feed materials from registered business entities, ban on using waste for feeding insects, conditions set out with respect to buildings and facilities in which insect farming can be conducted, organizational problems, the required thermal processing of frass for fertilization purposes, determination of the number of specimens, waterers, feeders or transport of products in authorized means of transportation.

### 3.9. Identified Legal Problems Affecting Insect Framing and Production of Fertilizer from Frass

**Figure 3:**
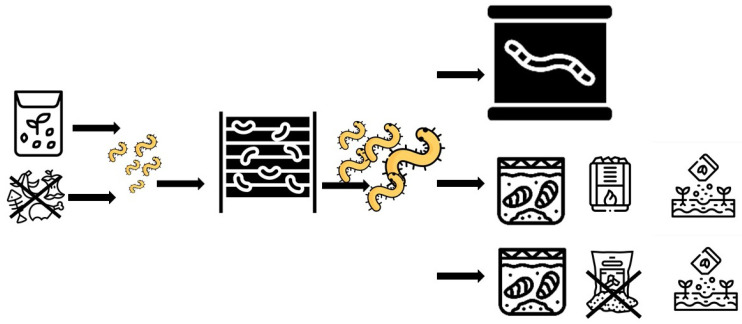
Identified legal problems in insect farming and production of insect products in Poland.

Following the analysis of the general issues suggested by stakeholders, a review of the legal regulations governing the preparation and production of frass was made. The assumptions for the case study submitted to our analysis related to the following stages: (1) insect rearing with the use of residues from agriculture, agricultural and food production industry and some other waste for insect farming; (2) creating a new product from a by-product obtained during insect rearing, i.e., frass-based fertilizer, i.e., a mixture of insect feces with feed and dead insect remains collected from the rearing of mealworms fed wheat (and other cereal) bran and oilseed press cake, used as raw materials for making feed. Under these assumptions, and based on the analysis of the binding legal regulations in this field, the following legal problems in the insect production chain were identified (Figure 3).

1. The scope of approvals for feeding insects with residues from agriculture, agriculture and food production industry, waste, and delicatessen and catering by-products;

2. Requirements imposed on the processing of frass into fertilizers used in agricultural and horticultural production; approval for sale.

### 3.10. Suggested Legal Solutions

#### 3.10.1. Introduction of New Raw Materials (Including Catering Waste) for Feeding Insects

At the production stage, the expansion of the insect production chain depends on solving the question of what materials can be used for feeding insects. Allowing insects to be fed with residues from agricultural farms or with catering waste would help to supplement two chains. One is the chain of managing agricultural production residues for the purpose of feeding insects during their rearing stage. The other chain of supplies refers to the possibility of using agricultural production residues and catering waste for insect nourishment, which would allow us to complement the chain of agricultural production residues by farmers and agricultural producers.

#### 3.10.2. Relaxing Constraints in Frass-Based Fertilizer Production

Because the EU and national law account for the specific character of insect farming and processing only to a small extent, amendments in certain areas are needed to address these particularities. Legal regulations should, therefore, be supplemented and adjusted to the technological, technical and organizational capacities of insect rearing and production of insect-based products. The proposed scope of changes in law aims to facilitate the fulfilment of legally binding procedures by businesses and to reduce the costs of insect production while respecting the existing legal procedures and technologies and without compromising the safety of people and animals. The suggested legal adjustments take advantage of the current organizational, financial and technical solutions applicable to insect farming and processing for feed, with the aim to reduce production costs.

It appears that, owing to these changes, the rearing of insects and production of insect-based products would be less expensive. The suggested modifications would primarily affect frass production, although the rearing of insects for other purposes could also be improved on the condition that all safety measures be maintained. This is one of the purposes of the adoption to the new regulation, Regulation No 2017/893 [39] amending Regulation No 999/2001 [29]. At present, insect rearing is conducted on a relatively small scale. The rearing of insects for animal feed could be adequately regulated within the framework of the existing national law, compliant with the EU law governing the health of animals, public health, health of plants or environmental risks. A proposed change in this regard is the approval of a method for frass processing that would be analogous to the processing of by-products from animal production envisaged in point G, Chapter III. Annex IV of Regulation of the Commission (EU) No 142/2011 [28]. Under this method, there is an option to implement any processing method authorized by the competent authority, provided the applicant has proven that the requirements laid down in this regulation have been met. Such changes would help producers to decrease frass production costs while maintaining safety measures.

## 4. Conclusions

The SWOT survey addressed to a group of people involved in insect rearing and the production of insect-based products (including frass) demonstrated both strengths and weaknesses of this branch. Most strengths referred to technological factors, where the innovative aspect of insect production was most often mentioned, while some of the weaknesses indicated by the respondents were technological and organizational difficulties as well as underdeveloped production technologies. Most of the external factors that affected the insect production industry, seen as either opportunities or threats, belonged to the sphere of economy. The opportunities were new markets in the feed and pet food sectors, whereas constraints were associated with competitive products made outside the EU, especially in Asia.

As demonstrated, the current legal framework is not optimal for insect farming for various purposes, including food or fertilizer production. One of the reasons is that the general legal regulations apply to all farmed animals, and no attention is paid to the unique nature of insect farming. However, even in the current situation, a few amendments could facilitate feed sourcing or the marketing of certain products. In view of the above, the following recommendations are proposed:

(1) Expansion of the catalogue of products used and allowed for insect nutrition by adding agricultural residues or food residues (catering waste), which would allow us to complement two chains (agricultural production and agricultural and food industry) while reducing costs of waste management by agricultural producers and catering establishments. In this regard, it could be recommended to expand the catalogue of plant substrates allowed for feeding insects listed in Section 10 of Annex X of Regulation (EC) No 142/2011 [37] by adding catering waste. Currently, what the mentioned annex allows is using the food that is no longer intended to be consumed by people, but that will be used as animal feed, or by-products obtained during food production or even food end-products but not catering waste. It is worth noting that under the present legal conditions, insects can be fed by-products but not waste. Therefore, only the waste that has been classified as by-products in the light of Article 5 of Directive 2008/98/EC [32] on waste can be used by insect breeders, which is quite a large barrier to insect farming, where there are many substrates that fail to satisfy this requirement. As the subject analyzed here is insect farming for feed and not for food, the requirements regarding the use of catering waste classified as waste, and not by-products, should be relaxed. Thus, the mentioned Annex X should directly list it as catering waste or residues from delicatessen production.

(2) A change in the hygiene requirements regarding production of frass for fertilization purposes laid down in Annexes I and X to Regulation of the Commission (EU) No 142/2011 [37]. Under the current legal conditions, one of the requirements defined by the European Commission in the field of microbiological safety is the need to submit frass to thermal sterilization at a temperature of at least 70 °C, and a sterilization process in a sterilizer should last for 60 min without interruptions for the product to meet the criteria set out in Regulation (EC) No 2021/1925 [44]. This, however, is costly and labor consuming. In order to reduce costs of frass preparation and processing for fertilization purposes, it would be recommendable to expand the catalogue of methods by introducing a solution analogous to the one applied to by-products from animal processing envisaged as method 7 in point G Chapter III of Annex IV Regulation (EC) No 142/2011 [37]. This provision allows for the possibility to implement any processing method authorized by the competent authority provided that a given business entity has proven that the said method satisfies the requirements specified in this regulation. Should a similar solution be accepted for production of frass, frass producers could opt for the most economically viable method. This would enable entrepreneurs to reduce frass production costs while maintaining the safety of the product.

This paper deals with the identification of legal challenges related to the production of insects for animal feeds in Poland; therefore, the scope of regulations concerning the marketing of insect products as food globally and in Poland was not analyzed in greater detail. However, to present some background information on farmed insects, the authors also referred to certain global tendencies in insect production for human nutrition purposes, underlying the significance of safety, which calls for specific legal regulations. This also applies to the production of insects for animal feed or the use of frass for the fertilization of crops. Moreover, when identifying the legal barriers to insect farming and processing, two main problems indicated by the workshop participants, who made up a relatively small group of respondents, were described. This, however, does not exclude the presence of other legal problems that can have a considerable impact on insect production and manufacture of insect-based products.

## Figures and Tables

**Table 1:** EU legal status of insect processing.

Areas of Regulation	Legal Acts	General Solutions
Insect rearing	Regulation (EC) No 178/2002 [30], 852/2004 [31], 853/2004 [32] and 2017/625 [33] Regulation (EC) No 1169/2011 [34] Regulation (EC) No 767/2009 Annex III [35]	Food and feed safety. HACCP system. Food labeling. Prohibits the use household waste or manure for animal nutrition.
Feeding of farmed insects	Regulation No 1069/2009 [36] Regulation (EC) No 142/2011 Annex IV [37] Regulation (EC) No 999/2002 Annex XIV Chapter 1 Section 2, 5b [38], Regulation (EC) No 1069/2009 [37], Regulation (EC) No 142/2011 [37] Regulation (EC) No 999/2001 Article 7 and Annex IV [38] Regulation (EC) No 68/2013 [39] Regulation (EC) No 767/2009 Annex III [35] Regulation (EC) No 1831/2003 [40] Regulation (EC) No 142/2011 Section 10 Annex X [37] Directive No 2002/32/EC [41] Directive No 2008/98/EC Article 5 [42] Regulation (EC) No 1069/2009 [36]	Classification of insects as farmed animals. Materials approved for insect nutrition. A ban on the use of manure, catering waste and untreated animal by-products. A feed ban. A catalogue of feed materials materials prohibited in animal nutrition. Authorized feed additives food removed from human consumption. Undesirable substances in animal feed. Insects intended as fishing bait. Recognition of a substance as a by-product. By-products for processing animal protein.
Insect species for feed production	Regulation (EC) No 999/2001 Article 7(1) [38]. Regulation (EC) No 767/2009 Article 4 [35]. Regulation (EC) No 1069/2009 Articles 10 and 31 [36], Regulation (EC) No 142/2011 Annex X [37]. Regulation (EC) No 142/2011 Annex X [37]. Regulation (EC) No 142/2011 Annex I [37]. Regulation (EC) No 1069/2009 [36]. Regulation (EC) No 142/2011 Chapter III Annex IV [37]. Regulation (EC) No 2017/893 [43]. Regulation (EC) No 2021/1925) [44].	A feed ban. Safety and marketing requirements. Fats intended to feed. The processing of fats and hydrolyzed proteins requirements. Products of animal origin that do not require processing such as PAPs. Three categories of animal by-products. Processing of insect biomass to PAPs. The lists of insect species approved for farming. Added species to the above list silkworm (*Bombyx mori*).
Regulation of feed production	Regulation No 183/2005 Article 2 [45]. Regulation No 183/2005 Annex II [45]. Regulation (EC) No 142/2011 Annex IV [37]. Regulation (EC) No 767/2009 [35]. Regulation (EC) No 178/2002 Article 15 [31].	Business entities operating on the feed market. Rules of food hygiene and an HACCP system. Methods using by insect-based feed manufacturers. Requirements applied to feeds sold on the market. Marking and labeling of feed.
Welfare of insects	Directive 98/58/EC Article 1 [46].	Welfare in animal rearing.
Frass as fertilizer	Regulation (EU) No 142/2011 Annex I [37]. Regulation (EU) 2021/1925 [44]. Regulation (EU) No 2019/1009 [47].	The possibility of using frass for fertilization. Safety requirements for frass to meet. Insect farming regulations.

**Table 2:** Polish legal status of insect processing.

Areas of Regulation	Legal Acts	General Solutions
Feeding of farmed insects	Act of 14 December 2012, on waste Article 10 [48].	Recognition of a substance as a by-product.
Insect production for feed	Act of 6 March 2018, Entrepreneurs’ Law Article 6 item 1 point 1 [49]. Act of 16 December 2005, on products of animal origin [50].	Requirements applied to business activity in the rearing and production of useable insects on a farm the procedure for the registration of business entities.
Frass as fertilizer	Act of 10 July 2007 on fertilisers and fertilisation [51] Regulation of the Minister of Agriculture and Rural Development of 9 August 2024 on implementation of certain provisions of the Act of Fertilisers and Fertilisation [52].	Insect farming regulations.

## Data Availability

Data are contained within the article.
